# Optical coherence tomography angiography as a potential tool in differential diagnosis of multiple sclerosis and rheumatic disorders with central nervous system involvement

**DOI:** 10.1007/s10792-024-03217-3

**Published:** 2024-06-26

**Authors:** Ewa Abecasis Fernandes, Paula Wildner, Magdalena Oset, Małgorzata Siger, Mariusz Stasiołek, Mariola Matysiak, Michał Wilczyński

**Affiliations:** 1 Department of Ophthalmology, Medical University of Lodz, 90-414 Lodz, Poland; 2 Department of Neurology, Medical University of Lodz, 90-414 Lodz, Poland

**Keywords:** Optical coherence tomography angiography, Multiple sclerosis, Rheumatic disorders, Differential diagnosis

## Abstract

**Purpose:**

The aim of this study is to analyse whether optical coherence tomography angiography (angio-OCT, OCTA) measurements can be a useful tool to differentiate central nervous system (CNS) involvement in rheumatic disorders (RD) from multiple sclerosis (MS).

**Methods:**

A total of 85 patients- 41 with MS, 21 with RD with CNS involvement and 23 healthy controls were included in the study. All individuals underwent OCTA and the following parameters were measured in each eye separately: average foveal and parafoveal vessel density (VD), average foveal and parafoveal vessel length (VL) of the superficial capillary plexus (SCP) and deep capillary plexus (DCP), as well as area, perimeter, and circularity of the foveal avascular zone.

**Results:**

OCTA showed a VD reduction in the foveal region of the SCP in eyes of RD patients when compared to MS patients (21.96 ± 3.39 vs.23.88 ± 3.05 (*p* = 0.003)).

There have been no significant differences in any of the assessed parameters that is average VD and total average VL in the foveal area of the SCP as well as of the DCP in the general population comprising healthy controls, MS and RD groups (*p* > 0.05 for all).

**Conclusions:**

Our results suggest that an OCTA finding of decreased VD in the foveal region of the SCP may be considered as a potentially useful biomarker of RD in comparison with MS patients.

## Introduction

For many years fluorescein angiography (FA) has been a gold standard of retinal and choroidal vasculature examination. This method is gradually being replaced by angio-OCT (OCTA). OCTA is a non-invasive, easier, faster and more economically reasonable method of examination than FA [1]. While FA requires an injection of fluorescent dye (sodium fluorescein) into the peripheral vein, which can cause nausea, emesis, syncope, allergic reactions and even anaphylactic shock to the patient, OCTA does not require any dye injection and is a very fast and repeatable examination [2]. Moreover, FA is not advisable for pregnant women, as well as patients with renal impairment and cardiological diseases [1]. OCTA detects the movement of erythrocytes in the blood vessels, due to which we can evaluate the shape, density and other measurable parameters of the blood vessels [1]. Thanks to OCTA, three dimensional and cross sectional images are obtained, which is another advantage over the two dimensional visualisation provided by FA [1].

OCTA is not only a tool in the diagnostic process of retinal pathologies such as age-related macular degeneration, central serous chorioretinopathy, diabetic retinopathy, vascular occlusions etc., but also a means to evaluate the perfusion of the optic disc [3]. With the use of OCTA, it is possible to detect the damage of the optic disc and monitor the progression in glaucoma [4]. Thanks to evaluation of the optic disc perfusion it has been possible to spot the abnormalities in neurodegenerative diseases such as multiple sclerosis (MS), Alzheimer´s disease, arteritic and non-arteritic optic neuropathy (AION and NAION), Parkinson´s disease, Leber’s hereditary optic neuropathy, among others [4].

Due to similarities between the vascular structures of retina and the brain, OCTA could potentially reflect pathological processes in the neurodegenerative diseases [5, 6]. In fact, optical micro-angiography of mice have been conducted for the study of brain injuries, and strokes [5].

Multiple sclerosis (MS) is considered to be the most frequent acquired demyelinating disorder of the central nervous system [7]. On the other hand, patients with autoimmune connective tissue diseases present a wide range of manifestations of central as well as peripheral nervous system injury [8]. Neurological involvement in autoimmune connective tissue diseases includes, among others, central nervous system demyelination, which implies the differential diagnosis of MS [8]. Studies have revealed that patients with MS, with as well as without prior history of optic neuritis, have a significantly diminished optic nerve head (ONH) perfusion, assessed as reduced ONH flow index, than healthy controls [9]. Other findings comprising patients with MS have shown an inverse correlation between vessel density (VD) parameters and the Expanded Disability Status Scale (EDSS) score, which reflects the level of disability [10]. Recently published studies have shown reduced retinal blood vessel density in the macular and peripapillary areas alone or simultaneously, in MS patients versus the control group [11–15].

In our previous study we concluded that SD-OCT cannot be incorporated in the differential diagnosis of multiple sclerosis (MS) and rheumatic disorders (RD) with CNS involvement. The study found a significant thinning of superior optic disc retinal nerve fiber layer (RNFL), macular RNFL, ganglion cell complex (GCC) thickness, ganglion cell layer-inner plexiform layer (GCIPL) thickness and smaller macular volume in MS patients versus healthy controls. However, no significant differences of the same parameters were observed between RD patients with CNS involvement [16].

Little is known about the role of OCTA in the diagnosis of rheumatic diseases with CNS involvement. Current research has revealed enlargement of the foveal avascular zone (FAZ) and a decrease of the parafoveal vessel density of deep capillary plexus (DCP) in patients with systemic lupus erythematosus (SLE) in comparison with healthy controls. Furthermore, an inverse correlation between vessel density parameters, namely parafoveal vessel density in DCP, and disease activity index in SLE has been found [].

Diagnosing and distinguishing between MS and RD with CNS involvement is currently very difficult and challenging but is a paramount importance for the selection of the appropriate treatment plan and follow-up for each patient. Nowadays there is no fully specific diagnostic test available. MRI examination, laboratory tests and clinical examination are required to ascertain the correct diagnosis, although results of magnetic resonance imaging (MRI) of the brain and spinal cord might be sometimes very confusing, because of overlapping of radiological findings in both groups.

The aim of this study was to investigate whether OCTA can be a useful tool to distinguish between multiple sclerosis and autoimmune connective tissue diseases with CNS involvement.

## Study design

We recruited patients diagnosed with MS (relapsing–remitting disease phenotype) and rheumatic disorders (RD) with CNS involvement to the study immediately after or during the diagnostic process in the Neurology Department of the Medical University of Lodz in Poland over a period of 16-months (July 2018–October 2019). The study was approved by the Local Ethics Committee of Medical University of Lodz -approval number RNN/231/18/KE, 12 June 2018.

2017 McDonald criteria were used to classified patients to MS group. All individuals underwent magnetic resonance imaging (MRI) and cerebrospinal fluid (CSF) examination during the diagnostic process. MRI examination confirmed in both groups (MS and RD) demyelinating-type lesions. We included patients from 18 to 50 years of age. All individuals were examined ophthalmologically—the examination included anamnesis, refraction test, best corrected visual acuity, tonometry, slit lamp examination with examination of anterior and posterior segment and pupil reactions of both eyes. Based on the examination we excluded patients with suspicion or confirmed diagnosis of glaucoma, retinal disorders, a refractive error exceeding ± 6 D, as well as opacities of the ocular optical media. The following criteria were used to eliminate eyes with the history of optic neuritis: sudden loss of visual acuity, pain during eye movements, loss of colour vision and improvement of vision after a few weeks. Moreover a visual evoked potentials test (VEP) have been performed for some patients during the diagnostic process. We considered that eyes with P100 amplitude reduction and P100 latency delay were excluded as suspected of optic neuritis. Healthy control subjects had negative history of neurologic and ophthalmologic diseases. After the process of enrolment, patients underwent the OCTA examination of the macula.

## Data collection

Each patient underwent OCTA measurements. They were performed with Copernicus REVO NX software version 9.5.0, Optopol Technology, Zawiercie, Poland.

**Fig. 1:**
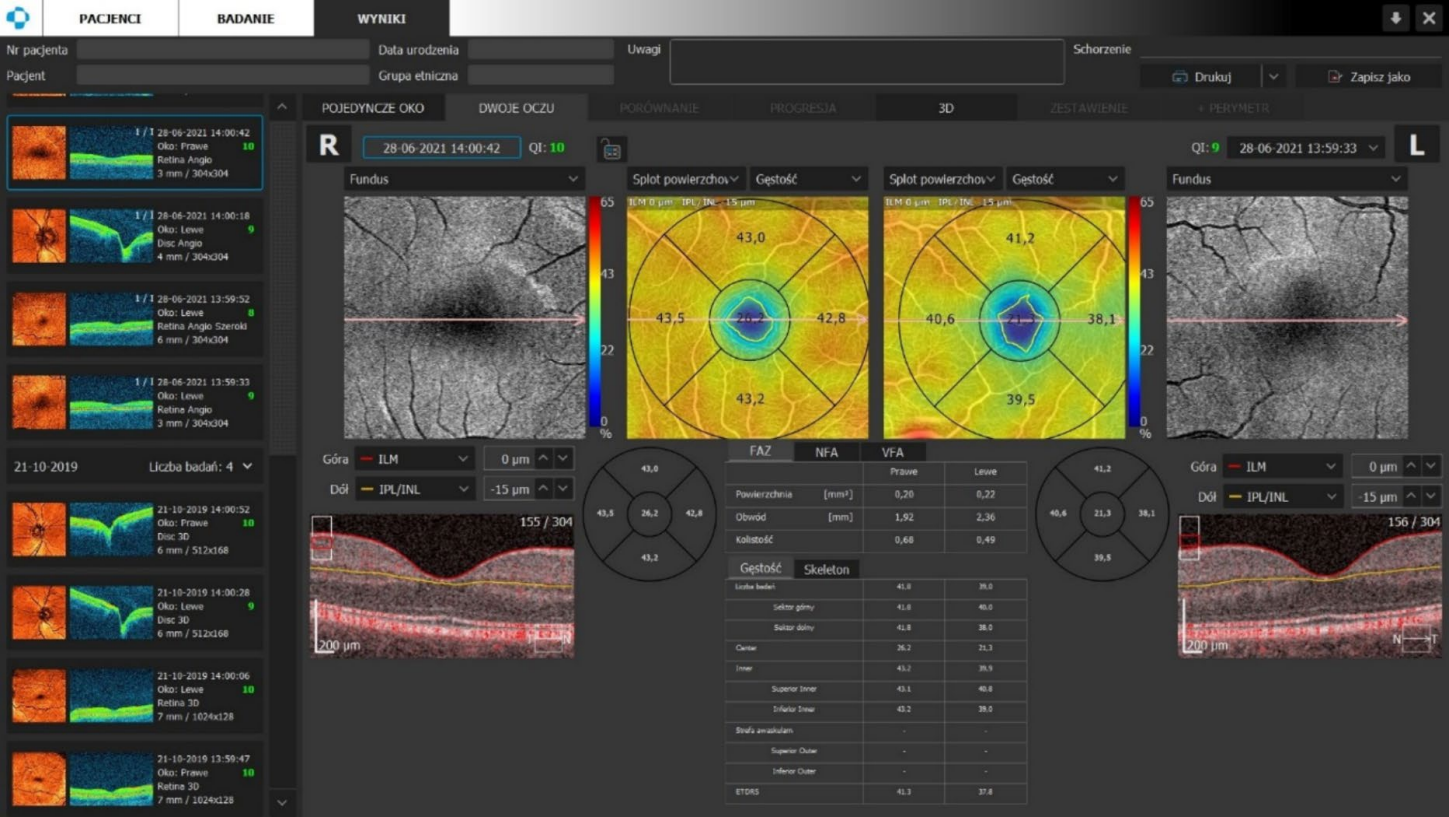
An example analysis of OCTA examination of the macula

Light source of REVO NX OCTA has wavelength of 830 nm. Scanning speed of this device is 110 000 measurements per second. An optical axial resolution is 5 microns, while transverse resolution is 12 microns. 3 × 3 mm scan mode (304 × 304 pixels) centred on the fovea was used to evaluate the macular capillary network. The superficial capillary plexus (SCP) and deep capillary plexus (DCP) images were segmented automatically by the software. Vessel density (VD) and vessel length (VL) in the fovea and in the parafoveal region in the superior, inferior, nasal and temporal sectors, as well as the FAZ area, perimeter, and circularity were automatically calculated by the in-built software. Measurements for parafoveal region were measured in for quadrants (superior, temporal, inferior and nasal) and an average for quadrants was calculated. In Fig. 1 an example analysis of OCTA examination of the macula.

OCTA examinations were performed by the same ophthalmologist. Scans with a low quality index (QI) below 8 or scans with artefacts, were excluded from the study.

## Statistical analysis

Statistical analysis was done using parametric tests. Statistical significance of differences between two groups with a confidence interval of 95%was determined using ANOVA test with a post-hocTukey test. All calculations were performed for the significance level α = 0.05 using Microsoft Excel and AddinsoftXLStat2008 software. A P value less than 0.05 was considered statistically significant.

## Results

### Participants characteristics and demographics

OCTA data were collected from 85 patients. They were divided into 3 groups: the first group—patients with MS (n = 41), the second group—patients with RD with CNS involvement (n = 21), and the third group—healthy controls (n = 23). Among patients from the first group, 9 patients besides multiple sclerosis had additional diseases in the history such as asthma, diabetes mellitus, Hashimoto and autoimmune hepatitis. RD with CNS involvement in the second group were the following: 5 patients with SLE, 6 patients with undifferentiated connective tissue disease, 2 patients with Sjogren syndrome, 2 patients with rheumatic arthritis, 2 patients with neurosarcoidosis, 1 with juvenile idiopathic arthritis and 3 with CNS vasculitis. All individuals underwent the diagnostic process in the Department of Neurology in the Medical University of Lodz. The brain MRI examination was performed in all participants. In MS patients MRI showed lesions fulfilling the MRI criteria included in 2017 McDonald diagnostic criteria. All individuals classified to the RD group presented demyelinating white matter lesions in the MRI examination. None of the patients from the RD group had history of immunomodulatory treatment. The mean age was 36.2 ± 8.47 years in group 1, 40.91 ± 8.52 years in group 2 and 39.91 ± 10.36 years in group 3-the differences were not significant (*p* > 0.05). In Table 1 demographic and clinical characteristics of the three groups are presented (Tables 2, 3, 4, 5, and 6).

**Table 1:** Study participants data

	G 1 MS	G 2 RD	G 3 HC
Patients, n	41	21	23
Eyes, n	70	42	46
Age (mean ± SD)	36.2 ± 8.47	40.91 ± 8.52	39.91 ± 10.36
Female, n	32	17	19
Male, n	9	4	4
Duration of neurological symptoms, (months; mean ± SD)	21.2 ± 26.8	48.2 ± 87.3	–

G1—group1, G2—group2, G3—group 3, *MS* Multiple sclerosis, *RD* Rheumatic disease, *HC* Healthy controls

**Table 2:** SCP vessel density

[mean + -SD]	G1 MS	G2 RD	G3 HC	p G1/G2	p G1/G3	p G2/G3
SCP density fovea	23.88 ± 3.05	21.96 ± 3.39	23.41 ± 3.83	0.01*	0.75	0.11
SCP density parafovea superior	42.09 ± 0.96	42.02 ± 1.81	41.88 ± 1.09	0.96	0.68	0.88
SCP density parafovea nasal	40.58 ± 1.28	40.69 ± 1.43	40.75 ± 1.49	0.92	0.79	0.97
SCP density parafovea inferior	41.41 ± 1.68	41.30 ± 4.60	41.50 ± 1.74	0.94	0.91	0.84
SCP density parafovea temporal	40.11 ± 1.24	40.07 ± 1.33	40.48 ± 1.27	0.98	0.28	0.29
SCP density parafovea average	41.05 ± 1.03	41.02 ± 1.31	41.16 ± 1.07	0.99	0.87	0.84

G1—group1, G2—group2, G3—group 3, *SCP* Superficial capillary plexus, *MS* Multiple sclerosis, *RD* Rheumatic disease, *HC* Healthy controls

An asterisk emphasizes that the *p* values (Tukey) are statistically significant

**Table 3:** SCP vessel length

[mean + -SD]	G1 MS	G2 DR	G3 HC	p G1/G2	p G1/G3	p G2/G3
SCP length fovea	16.93 ± 1.94	15.34 ± 2.51	16.06 ± 2.82	0.01*	0.13	0.33
SCP length parafovea superior	24.10 ± 1.74	23.31 ± 2.35	23.60 ± 2.51	0.01*	0.02*	0.80
SCP length parafovea nasal	24.48 ± 1.58	23.50 ± 2.02	23.56 ± 2.33	0.03*	0.04*	0.98
SCP length parafovea inferior	24.14 ± 1.75	23.22 ± 2.15	23.50 ± 2.71	0.08	0.27	0.82
SCP length parafovea temporal	24.15 ± 1.47	23.06 ± 2.09	23.43 ± 2.20	0.01*	> 0.11	0.62
SCP length parafovea average	24.37 ± 1.55	23.27 ± 2.08	23.50 ± 2.37	0.01*	0.06	0.82

G1—group1, G2—group2, G3—group 3, *SCP* Superficial capillary plexus, *MS* Multiple sclerosis, *RD* Rheumatic disease, *HC* Healthy controls

An asterisk emphasizes that the *p* values (Tukey) are statistically significant

**Table 4:** DCP vessel density

[mean + -SD]	G1 MS	G2 RD	G3 HC	p G1/G2	p G1/G3	p G2/G3
DCP density fovea	34.69 ± 2.31	34.03 ± 2.05	34.81 ± 2.55	0.32	0.95	0.26
DCP density parafovea superior	43.80 ± 1.50	43.88 ± 0.48	43.79 ± 0.47	0.92	0.99	0.92
DCP density parafovea nasal	43.76 ± 0.56	43.98 ± 0.51	43.90 ± 0.54	0.11	0.39	0.78
DCP density parafovea inferior	43.61 ± 0.94	43.75 ± 0.66	43.75 ± 0.80	0.66	0.64	1.00
DCP density parafovea temporal	43.71 ± 0.62	43.87 ± 0.48	43.90 ± 0.47	0.30	0.18	0.97
DCP density parafovea average	43.72 ± 0.58	43.87 ± 0.39	43.83 ± 0.41	0.27	0.44	0.94

G1—group1, G2—group2, G3—group 3, *DCP* Deep capillary plexus, *MS* Multiple sclerosis, *RD* Rheumatic disease, *HC* Healthy controls

**Table 5:** DCP vessel length

[mean + -SD]	G1 MS	G2 RD	G3 HC	p G1/G2	p G1/G3	p G2/G3
DCP length fovea	24.02 ± 1.74	22.94 ± 2.22	23.27 ± 2.43	0.02*	0.15	0.74
DCP length parafovea superior	26.55 ± 1.66	25.62 ± 2.42	25.60 ± 2.77	0.09	0.07	0.99
DCP length parafovea nasal	25.83 ± 1.49	24.95 ± 2.09	25.02 ± 2.37	0.06	0.07	0.98
DCP length parafovea inferior	26.12 ± 1.57	25.06 ± 2.29	25.43 ± 2.67	0.03*	0.20	0.69
DCP length parafovea temporal	25.64 ± 1.44	24.55 ± 2.03	24.81 ± 2.40	0.02*	0.06	0.92
DCP length parafovea average	26.04 ± 1.48	25.07 ± 2.17	25.21 ± 2.51	0.04*	0.08	0.94

G1—group1, G2—group2, G3—group 3, *DCP* Deep capillary plexus, *MS* Multiple sclerosis, *RD* Rheumatic disease, *HC* Healthy controls

An asterisk emphasizes that the *p* values (Tukey) are statistically significant

**Table 6:** FAZ parameters

[mean + -SD]	G1 MS	G2 RD	G3 HC	p G1/G2	p G1/G3	p G2/G3
FAZ area	0.23 ± 0.08	0.24 ± 0.08	0.24 ± 0.10	0.59	0.81	0.94
FAZ perimeter	2.23 ± 0.40	2.27 ± 0.37	2.21 ± 0.52	0.90	0.97	0.82
FAZ circularity	0.57 ± 0.12	0.59 ± 0.11	0.57 ± 0.12	0.62	0.97	0.78

G1—group1, G2—group2, G3—group 3, *FAZ* Foveal avascular zone, *MS* Multiple sclerosis, *RD* Rheumatic disease, *HC* Healthy controls

### Comparison of SCP vessel density

The study showed a significant difference in average vessel density (VD) in the superficial capillary plexus (SCP) in the foveal area (*p* = 0.003) between the MS group and the RD group. The RD group had lower average VD values than the MS group in the foveal region (21.96 ± 3.39 vs. 23.88 ± 3.05). No significant differences in the average VD in the foveal area were revealed between the patients from the MS/ and RD groups, and the control group (*p* ˃ 0.05). Moreover, no significant differences in the average VD in the parafoveal area were found between all three groups (*p* ^˃^ 0.05 for all)(Table 2).

### Comparison of SCP vessel length

The total average VL of SCP in the foveal region was significantly lower (*p* = 0.0003) in the RD group when compared to MS group (15.34 ± 2.51 vs. 16.93 ± 1.94). No significant differences in the total average VL in the foveal area were revealed between the patients from the MS and RD groups, and the control group (*p *˃ 0.05). In the parafoveal region the MS group’s total average VL was longer than both the RD and control groups (MS vs. RD 24.37 ± 1.55 vs. 23.27 ± 2.08; *p* = 0.002 and MS vs. controls 24.37 ± 1.55 vs. 23.52 ± 2.37; *p = 0.022*)(Table 3)*.*

### Comparison of DCP vessel density

No significant differences were observed in the vessel density in the deep capillary plexus (DCP) among all three groups in the foveal and parafoveal area (*p* ^˃^ 0.05 for all) (Table 4).

### Comparison of DCP vessel length

The total average VL of DCP in the foveal region was significantly lower in the RD group when compared to the MS group (22.94 ± 2.22 vs. 24.02 ± 1.74) while there were no significant differences between controls and both patients groups (MS and RD group) in this parameter. No significant differences were observed in the total average VL of DCP in the parafoveal region among all the three groups (*p* ^˃^ 0.05 for all)(Table 5).

### Comparison of FAZ area, circularity, and perimeter

No significant differences in the calculated FAZ area, circularity and perimeter were identified between all three groups (*p* ^˃^ 0.05 for all) (Table 6).

## Discussion

Accurate diagnosis of MS is often very challenging. It is crucial to make a proper diagnose to initiate a necessary therapy which varies, depending on the cause. It has been reported so far that SD-OCT is not a specific biomarker to distinguish between patients with MS and RD with CNS involvement [16].

Our current results imply that an OCTA finding of decreased VD in the foveal region of the SCP may be regarded as a potentially useful biomarker of RD in comparison with MS patients. However, there have been no significant differences in any of the assessed parameters that is average vessel density and total average vessel length in the foveal area of the SCP as well as of the DCP in the general population comprising healthy controls, MS and RD groups. OCT has long been involved in the examination of MS patients. However, data comprising OCTA is MS patients are scarce. Interestingly our study did not demonstrate significant differences of foveal and parafoveal VD in both SCP and DCP between MS patients and healthy controls. The results of currently available studies are not consistent. According Feucht et al. [17] found that patients with MS with history of optic neuritis (ON) have lower VD in SCP and DCP compared to healthy controls. Cennamo and colleagues [18] found that relapsing–remitting multiple sclerosis patients without former optic neuritis had a lover VD in superficial capillary plexus compared to healthy controls. Moreover, another study also found a reduction of macular capillary perfusion in both groups MSON—and MSON + [19].

Recent studies involving patients with RD have revealed several distinctive features assessed with the use of OCTA in comparison with healthy controls. Nevertheless, to our knowledge, there had been no previous data on the potential use of OCTA as a biomarker in differential diagnosis of patients with white matter lesions of the CNS.

Our study has revealed a significant decrease in the average VD values in the foveal region in the RD group in contrast to the MS. Other research has shown that patients with systemic lupus erythematosus have a decrease in the VD of the superficial capillary plexus on OCTA, as well as an increase in the iris vessels flow, and enlargement of the FAZ [20].

Interestingly, current review of studies investigating early pathological changes in retinal vascularization examined with OCTA presented that seven out of seven papers found in this field have revealed a decrease in SCP in ocular asymptomatic patients diagnosed with SLE. Thus, it has been postulated that a decrease in retinal vessel density measured by OCTA may be a good marker of SLE activity and poor prognosis [21].

Remarkable differences have been found between patients with systemic sclerosis and healthy controls regarding vessel density in the DCP. Study by Carnevali and colleagues has shown significantly lower DCP VD in the whole scan and in the perifoveal, superior, inferior, nasal and temporal regions in that patients with systemic sclerosis. Moreover, a qualitative analysis of OCTA has detected at least one abnormality in 95% of patients with systemic sclerosis [22].

Another study comprising patients with RD, i.e. sarcoidosis, has shown discordant results. Unlike SLE, in patients with non-ocular sarcoidosis increased SCP and DCP vessel densities, in whole, parafoveal, and perifoveal, have been found in comparison with healthy controls [23].

In contrast, in patients with rheumatoid arthritis, retinal capillary plexus density in the macula, obtained from the SCP, DCP, and radial peripapillary capillary, is lower than in healthy controls [24].

This study shows that patients with RD with CNS involvement have significantly lower VD in the foveal region in the SCP than patients with MS. Capillaroscopy is a well-known, non-invasive and safe examination used in screening and follow-up of patients with RD. It can evaluate the morphological changes in the capillaries in the nailfold. A principal indication of capillaroscopy is among others distinction between primary Raynaud’s phenomenon (RP) and secondary RP, Scleroderma spectrum disorders—group of connective tissue diseases connected to systemic sclerosis (SSc) [25].

Morphological changes present in the vasculature of patients with RD include capillary loss, capillary shape changes, haemorrhages, avascular areas [26].

In our study we analysed FAZ metrics such as area, perimeter and circularity between all three groups. We found no significant differences in all the parameters between the groups. A recent study compared FAZ parameters between MS patients and healthy controls revealing no significant differences between both groups [27]. An association between enlarged FAZ area/declined circularity and the severity of diabetic retinopathy has been discovered. Moreover, it has been demonstrated that declined circularity is correlated with decreased visual acuity [28]. Microvascular damage was assumed by the authors as a possible reason for those alterations.

One of the chronic, rheumatic disease is SLE, in which the frequency of the retinopathy varies from 7 to 26%, based on activity and control of the disease [29]. The majority of the previous analysed studies has revealed the effect of SLE on the enlargement of FAZ area [19]. However, according to some studies no statistically significant differences were found in FAZ parameters between SLE patients and control groups [30, 31].

There were some limitations of our study. First of all, the group of RD with CNS involvement patients was small, because of the low prevalence compared to MS patients. Moreover, the RD group was very heterogenous. Patients with other co-morbidities have been included in the multiple sclerosis group which potentially could alter the results. For that reason, further investigation with larger cohort is needed to confirm our observations. Nevertheless, OCTA can be a useful tool in differential diagnosis process between RD patients with CNS involvement and MS.

## Conclusions

Our results suggest that a decreased vessel density in the foveal region of the superficial capillary plexus (SCP) may be considered as a potentially useful differentiating factor of rheumatoid diseases in comparison with multiple sclerosis patients.

Therefore, optical coherence tomography may be a useful tool in evaluating the retinal circulation in diseases characterized by CNS involvement.

## Abbreviations

FA: Fluorescein angiography
OCTA: Optical coherence tomography angiography
CNS: Central nervous system
MS: Multiple sclerosis
AION: Arteriticoptic neuropathy
NAION: Non-arteritic optic neuropathy
ONH: Optic nerve head
EDSS: Expanded Disability Status Scale
RNFL: Retinal nerve fiber layer
GCC: Ganglion cell complex
GCIPL: Ganglion cell-inner plexiform layer
FAZ: Foveal avascular zone
DCP: Deep capillary plexus
SLE: Systemic lupus erythematosus
RD: Rheumatic disorders
MRI: Magnetic resonance imaging
CSF: Cerebrospinal fluid
SCP: Superficial capillary plexus
VD: Vessel density
VL: Vessel length
QI: Quality index
RP: Raynaud’s phenomenon
SSc: Systemic sclerosis
ON: Optic neuritis
SD-OCT: Spectral domain optical coherence tomography
